# The Impact of the *FKBP5* Gene Polymorphisms on the Relationship between Traumatic Life Events and Psychotic-Like Experiences in Non-Clinical Adults

**DOI:** 10.3390/brainsci11050561

**Published:** 2021-04-28

**Authors:** Filip Stramecki, Dorota Frydecka, Łukasz Gawęda, Katarzyna Prochwicz, Joanna Kłosowska, Jerzy Samochowiec, Krzysztof Szczygieł, Edyta Pawlak, Elżbieta Szmida, Paweł Skiba, Andrzej Cechnicki, Błażej Misiak

**Affiliations:** 1 Department of Psychiatry, Wroclaw Medical University, Pasteur Street 10, 50-367 Wroclaw, Poland; fstramecki@gmail.com (F.S.); dfrydecka@gmail.com (D.F.); 2 Clinical Neuroscience Lab, Institute of Psychology, Polish Academy of Sciences, Jaracza Street 1, 00-378 Warsaw, Poland; lgaweda@wum.edu.pl; 3 Institute of Psychology, Jagiellonian University, Ingardena 6 Street, 30-060 Krakow, Poland; katarzyna.prochwicz@uj.edu.pl (K.P.); joanna.klosowska@uj.edu.pl (J.K.); 4 Department of Psychiatry, Pomeranian Medical University, Broniewskiego 26 Street, 71-457 Szczecin, Poland; samoj@pum.edu.pl (J.S.); kf.szczygiel@gmail.com (K.S.); 5 Department of Experimental Therapy, Hirszfeld Institute of Immunology and Experimental Therapy, Polish Academy of Sciences, Weigla Street 12, 53-114 Wroclaw, Poland; edyta.pawlak@hirszfeld.pl; 6 Department of Genetics, Wroclaw Medical University, Marcinkowskiego 1 Street, 50-368 Wroclaw, Poland; e.szmida@gmail.com (E.S.); pawel.skiba@umed.wroc.pl (P.S.); 7 Department of Community Psychiatry, Medical College Jagiellonian University, Sikorskiego Place 2, 31-115 Krakow, Poland; acechnicki@interia.pl

**Keywords:** schizophrenia, genetics, cortisol, HPA axis

## Abstract

Common variations of the *FKBP5* gene are implicated in psychotic disorders, by modulating the hypothalamic–pituitary–adrenal axis reactivity to stress. It has been demonstrated that some of them might moderate the effects of childhood trauma on psychosis proneness. However, these associations have not been investigated with respect to traumatic life events (TLEs). Therefore, we aimed to explore whether the *FKBP5* polymorphisms moderate the effects of TLEs on the level of psychotic-like experiences (PLEs). A total of 535 non-clinical adults were approached for participation, and genotyping of six *FKBP5* polymorphisms (rs3800373, rs9470080, rs4713902, rs737054, rs1360780 and rs9296158) was performed. The Prodromal Questionnaire-16 (PQ-16) and the Traumatic Events Checklist (TEC) were administered to assess PLEs and TLEs, respectively. Among the rs1360780 CC homozygotes, a history of physical abuse was associated with significantly higher PQ-16 scores. This difference was not significant in the rs1360780 T allele carriers. Similarly, a history of physical abuse was associated with significantly higher PQ-16 scores in the rs9296158 GG homozygotes but not in the rs9296158 A allele carriers. Finally, emotional neglect was related to significantly higher PQ-16 scores in the rs737054 T allele carriers but not in the rs737054 CC homozygotes. The present study indicates that variation in the *FKBP5* gene might moderate the effects of lifetime traumatic events on psychosis proneness.

## 1. Introduction

In recent years, a growing body of studies focus on the role of gene–environment interactions in the development of numerous mental disorders, including schizophrenia [1]. Traumatic life events (TLEs) have been considered a significant risk factor for the development of psychosis [2] and cognitive impairments in patients with schizophrenia [3], as well as cognitive biases [4] and psychotic-like experiences (PLEs) in non-clinical subjects [5,6]. Moreover, TLEs play a pivotal role in the pathophysiology of various mental disorders, including schizophrenia [2,3,7]. Although it has been reported that childhood traumatic experiences may lead to the development of psychosis [8], it has also been observed that cumulative lifetime trauma exposure has a significant influence on a risk of psychosis [9].

Notably, PLEs are considered one of the phenomena that lie on the continuum of psychosis, where non-clinical psychotic symptoms precede the onset of overt psychosis [10]. These experiences include bizarre experiences, perceptual abnormalities (e.g., hearing unusual sounds such as clicking, humming or ringing) and delusional-like ideas (e.g., persecutory ideations or magical thinking) that range from perceptual illusions to subclinical attenuated positive symptoms [5]. During the past two decades, the body of research on PLEs has systematically grown [11,12,13,14]. It was repeatedly reported that PLEs are common in the general population [15,16,17,18] and are often not associated with severe distress or a lack of insight [16]. The recent cross-national analysis based on more than 31,000 respondents in 18 counties estimated the prevalence rate of PLEs at 7.2% [19]. Moreover, it has been reported that individuals exposed to childhood trauma (especially emotional or sexual abuse) are more likely to experience PLEs [20]. Moreover, early life traumatic experiences increase the prevalence of PLEs in young adults [21], some of whom may even experience frequent hallucinatory and delusional experiences [22].

Exposure to acute and/or chronic stress alters proper functioning of the main stress hormone system—the hypothalamic–pituitary–adrenal (HPA) axis [23,24], and activates a cascade of biological interactions that increase a risk of psychosis [25,26]. The HPA axis response can be modulated by the FK-506 binding protein 5, encoded by the *FKBP5* gene located on the chromosome 6p21 [27]. The FKBP5 is a co-chaperone of the heat shock protein hsp90, which modulates the glucocorticoid receptor (GR) sensitivity to the main stress hormone—cortisol [27]. The interaction of the FKBP5 protein with the GR leads to decreased receptor affinity and entails suppressed nuclear translocation [28,29]. This interaction indicates that stress exposure, by causing an increase in the cortisol level, leads to up-regulated *FKBP5* expression and reduced GR activity [27]. It has been shown that patients with psychosis present increased expression of *FKBP5* mRNA in the dorsolateral prefrontal cortex [30] and the hippocampus [31] when compared to healthy controls. Hence, altered expression of the *FKBP5* gene may be correlated with the HPA axis dysregulation. It has been shown that patients with schizophrenia [32] and first-episode psychosis [33] present elevated blood levels of circulating cortisol when compared to healthy controls. Moreover, the cortisol awakening response in patients with schizophrenia is significantly flattened when compared to healthy controls [34]. Meta-analysis investigating the HPA axis response to experimental social stress revealed that patients with psychosis have lower cortisol levels both in anticipation and after exposure to social stress [35]. It has also been observed that individuals at ultra-high risk of psychosis present significantly higher salivary cortisol levels than healthy controls [36].

In the past two decades, numerous studies have focused on genetic polymorphisms and epigenetic modifications that may influence the HPA axis reactivity to stress [37]. It has previously been reported that four single nucleotide polymorphisms (SNPs), including rs1360780, rs3800373, rs9296158 and rs9470080, are associated with decreased sensitivity of GR to circulating cortisol, leading to diminished negative feedback of the HPA axis [38,39]. The *FKBP5* gene contains several polymorphic sites that may affect stress response, and thus a risk of psychosis [39]. It has been reported that specific SNPs of the *FKBP5* gene may have an impact on the severity of psychotic symptoms in patients with psychosis after adjustment for exposure to TLEs [38,39]. Indeed, the study by Mihaljevic et al. [39] revealed that the rs3800373 G allele carriers had presented a higher risk of schizophrenia after accounting for childhood trauma exposure than the TT homozygotes. However, individual SNPs of the *FKBP5* gene may also play a role in the development of PLEs in a non-clinical population [40,41,42]. Accordingly, the rs13860780 T allele carriers have been shown to present higher levels of positive and negative PLEs after exposure to childhood abuse [43,44]. A similar relationship was observed for the rs92961558 polymorphism in non-clinical young adults exposed to bullying in childhood, where the A allele was positively correlated with the level of PLEs [45]. It has been observed that the rs3800373 C allele carries exposed to trauma present decreased anxiety sensitivity [46]. Moreover, the rs4713902 C allele carries have been found to show higher baseline cortisol level than the rs4713902 TT homozygotes [47]. In turn, the rs737054 polymorphism is located within a highly conserved region of the *FKBP5* intron 5 that has high regulatory potential [48,49].

So far, studies investigating interactions of the *FKBP5* gene with stress exposure in individuals with psychosis and PLEs have mostly focused on childhood trauma experience [41,45,46,50]. Therefore, in this study we aimed to investigate the influence of the *FKBP5* gene polymorphisms on the association between the level of PLEs and lifetime exposure to stress.

## 2. Materials and Methods

### 2.1. Participants

The sample included 535 individuals aged 18 to 30 (23.4 ± 3.0 years) recruited from university students of various faculties (computer science, mathematics, medicine, nursing, pedagogy and psychology) from three big cities in Poland (Krakow, Wroclaw, and Szczecin). All participants represented Caucasian ethnicity and were non-consanguineous. A history of clinical diagnosis was provided with a self-report questionnaire designed for the study. The Ethics Committee at Wroclaw Medical University (Wroclaw, Poland) approved the study protocol, and all participants gave written informed consent (project number: STM C230.018.34; approval number: 254/2018; issued on 19 July 2018).

### 2.2. Measures

#### 2.2.1. The Traumatic Events Checklist (TEC)

The TEC was used to assess a history of TLEs [51]. It is a self-report questionnaire that consists of 29 items. To measure emotional neglect (EN) we used the item: “When you were a child or a teenager have you ever felt emotionally neglected (e.g., being left alone, insufficient affection) by your parents, brothers or sisters?”. Emotional abuse (EA) was assessed with the use of the item: “When you were a child or a teenager have you ever felt emotionally abused (e.g., being belittled, teased, called names, threatened verbally, or unjustly punished) by your parents, brothers or sisters?”. Physical abuse (PA) and bullying was evaluated with the item: “When you were a child or teenager, did you experience physical abuse (e.g., tormenting, beating, psychically hurting) from your parents, brothers or sisters or peers?”. Sexual abuse (SA) was measured with the item: “When you were a child or a teenager have you ever been sexually harassed or abused by your parents, brothers or sisters or strangers?”.

#### 2.2.2. The Prodromal Questionnaire 16 (PQ-16)

The PQ-16 is a 16-item self-report questionnaire screening for psychosis risk and the presence of PLEs [52]. It consists of items assessing experiences of positive symptoms (nine items investigating perceptual aberrations as well as five items screening for delusional ideation, unusual thought content and paranoia) and two items focusing on negative symptoms. The original questionnaire consists of two scales, where the first investigates PLEs presence by “present” and ”non-present”; the second measures associated emotional distress by a four-point Likert scale. We used the Polish version of PQ-16, which was developed with the use of a back-translocation procedure and also was used in our previous studies [53]. In the present study, the level of distress associated with experiencing PLEs, further referred to as the PQ-16 score, was used as the outcome variable. Considering that perceptual abnormalities and delusional ideas are the first anomalies that can lead to psychosis development, we excluded items “1” and “7”, which investigate negative symptoms.

### 2.3. Genotyping

In the present study, we selected six SNPs (rs3800373, rs9470080, rs4713902, rs737054, rs1360780 and rs9296158) based on their functional impact on the *FKBP5* gene and the HPA axis activity. DNA samples were obtained using buccal swabs and the prepIT•L2P kit (DNA Genotek, Ottawa, ON, Canada). Although blood is usually collected to obtain DNA (it provides not only nucleated cells containing DNA but also many other physiological factors contained in plasma), the alternative, noninvasive sampling methods based on cheek-cell collection (oral or buccal epithelial cells collected with swabs, brushes or mouthwashes) are recommended in cases of large, population-based and multicentric studies [54]. Preference of buccal cells to obtain DNA is also related to unavailability of medical staff required to collect blood, and provides sufficient DNA quantity and quality. It should also be noted that buccal swabs are less contaminated by proteins compared to other methods of collecting oral biological material, and thus they enable improved quality and quantity of DNA [54,55]. Six common SNPs of the *FKBP5* gene (rs3800373, rs9470080, rs4713902, rs737054, rs1360780 and rs9296158) were genotyped with the allelic discrimination technique using validated and predesigned TaqMan^®^SNP Genotyping Assays (C__27489960_10, C_____92160_10, C__30559929_10, C___1256778_10, C___8852038_10, and C___1256775_30, respectively) according to the manufacturer’s instructions (ThermoFisher Scientific Inc., Waltham, MA, U.S.). In accordance with current recommendations for buccal cell collection, we decided to perform genotyping in duplicates for 25% of randomly selected samples to control for genotyping accuracy [56,57]; we decided to control our results (25% of randomly chosen samples from both groups) to check for genotyping accuracy. The results were controlled (25% of randomly chosen samples from both groups) to check for genotyping accuracy. Identical genotypes were identified in all duplicates. Subjects involved in genotyping were blinded to ID of participants and the data collected by specific questionnaires used in this study.

### 2.4. Statistics

The χ^2^ test was used to assess whether the distribution of genotypes followed the Hardy–Weinberg equilibrium (HWE). Bivariate comparisons were performed using the Mann–Whitney U test. The analysis of covariance (ANCOVA) was performed to test the effects of specific TLEs, SNPs and interactions between TLEs and SNPs on the PQ-16 score. Age and gender were added as the covariates. Separate models for specific SNPs and TLEs were tested. Post hoc comparisons were performed using the Games–Howell test in case of significant two-way interactions. Due to multiple testing, the Benjamini–Hochberg correction with the false discovery rate of 25% was applied. After this correction, results of all tests were considered significant if the *p*-value was less ≤ 0.022.

## 3. Results

Main characteristics of all participants are presented in Table 1. Out of 535 individuals approached for participation, 461 individuals provided data on a history of TLEs and the level of PQ-16 (86.2%). Sufficient quality of DNA was obtained for 441–449 participants (82.4–83.9%). Rates of EN, EA, PA and SA were as follows: 34.1%, 41.4%, 15.4%, and 8.2% participants, respectively. As expected, a history of all categories of TLEs was associated with significantly higher PQ-16 scores (Table 2). Clinical diagnosis (mood and anxiety disorders) was reported by 8.2% of the sample. None of these participants reported being diagnosed with psychotic disorders.

Main and interactive effects of the *FKBP5* SNPs on the PQ-16 score are shown in Table 3. There were significant effects of interactions between PA and two *FKBP5* SNPs (rs1360780 and rs9296158) on the PQ-16 score. Similarly, the interaction between EN and the rs737054 polymorphism was significantly associated with the PQ-16 score. In the majority of models, significant main effects of age and TLEs were found. In two models, main effects of the rs3800373 (the model with EA) and the rs9296158 (the model with PA) were observed.

**Figure 1:**
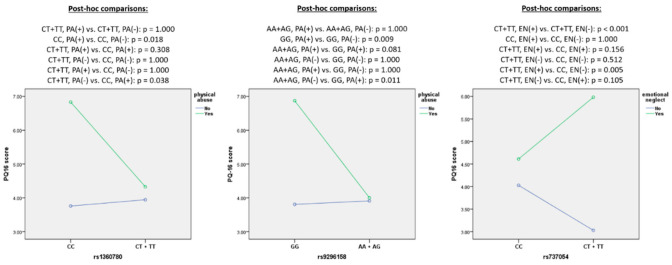
Interactive effects of the *FKBP5* genotype and TLEs on the PQ-16 score.

Results of post hoc analyses are presented in Figure 1. Among the rs1360780 CC homozygotes, a history of PA was associated with significantly higher PQ-16 scores. This difference was not significant in the rs1360780 T allele carriers. Similar findings were observed for the rs9296158 polymorphism. Indeed, a history of PA was associated with significantly higher PQ-16 scores in the rs9296158 GG homozygotes. The rs9296158 GG homozygotes reporting a history of PA had also significantly higher PQ-16 scores in comparison with the rs9296158 A allele carriers without a history of PA. Finally, EN was related to significantly higher PQ-16 scores in the rs737054 T allele carriers but not in the rs737054 CC homozygotes. The rs737054 T allele carriers had significantly higher PQ-16 scores with a history of EN in comparison with the rs737054 CC homozygotes who did not report EN.

## 4. Discussion

Results of this study support previous findings from studies testing the moderating effects of the *FKBP5* gene polymorphisms on the association between trauma exposure and a risk of psychosis or PLEs. More specifically, we found that a history of PA increases a severity of PLEs in the rs1360780 CC and the rs9296158 GG homozygotes. This may be explained by the role of the rs1380780 polymorphism in inducing the *FKBP5* gene transcription in response to GR activation [58] followed by stronger cortisol reactivity in response to stress [59] in individuals exposed to trauma carrying the “risk” T allele. Disinhibited induction of the *FKBP5* mRNA is responsible for GR resistance and causes diminished negative feedback of the HPA axis, leading to its dysregulation [60]. This stays in line with previous research supporting the role of the rs1360780 and rs9296158 in moderating the effects of childhood trauma on the development of positive PLEs [43,44,45]. However, these studies have reported that carries of rs1360780 T allele are more prone to develop psychotic symptoms [44] or greater subclinical psychotic symptoms [49] after exposure to childhood trauma, while we observed the opposite association, where the C allele was associated with greater severity of PLEs in response to PA. These mixed findings may be associated the fact that previous studies focused only on childhood trauma, while we assessed lifetime TLEs. Interestingly, Yaylac et al. observed that the rs1380780 C allele carriers and the rs9296158 G allele carriers exposed to childhood maltreatment develop significantly more severe dissociative symptoms when compared to traumatized subjects carrying the rs1380790 T allele and the rs9296158 A allele, respectively [61]. In turn, dissociation has been associated with the development of overt psychosis and PLEs [62,63]. The study by Mitjans et al. investigated the effect of the *FKBP5* gene polymorphisms on treatment outcome in patients with schizophrenia showing that TT homozygotes for the rs13860780 polymorphism have higher risk of non-response to clozapine than the C allele carries [64]. Previous studies have proposed that compound with the ability to interact with FKBP5 could be beneficial in the treatment of stress-related disorders [57,65,66]. Taking into account the body of studies supporting the role of *FKBP5* in pathophysiology of stress-related disorders, including schizophrenia, future studies could consider the *FKBP5* gene as a potential target for the treatment of psychosis.

The present study also demonstrated that the rs737054 T allele is associated with a higher severity of PLEs in subjects exposed to EN. The effect of the rs737054 polymorphism on the development of PLEs has not been widely addressed. The only study investigating its role in the development of PLEs in response to childhood trauma failed to find significant associations [39]. Moreover, there are only two studies examining this SNP. One study did not confirm that the rs737054 polymorphism affects susceptibility to borderline personality disorder after considering the role of childhood trauma [67]. In turn, it has been shown that male carries of the T allele at this SNP, exposed to childhood trauma, present significantly greater anxiety sensitivity when compared to the CC homozygotes [46], suggesting the role of the rs737054 polymorphism in stress response by modulating the HPA axis reactivity. Our results suggest that there is an association between variants of the *FKBP5*, lifetime traumatic events and risk of psychosis. The mechanism of genetic variability influencing psychosis development in response to stress remains unclear. It has also been shown that neurotrophic factors, including the brain-derived neurotrophic factor (BDNF), responsible for neuroplasticity in the human brain, plays a moderating role in the development of psychosis [1,68] and PLEs [69] in individuals exposed to psychosocial stress. Despite multiple observations suggesting that gene–environment interactions may be responsible for individual differences in response to TLEs, further studies are required to understand the exact mechanisms underlying the effects of interactions between genes regulating response to stress and neuroplasticity on the risk of psychosis.

The present study has several methodological limitations that should be taken into consideration when interpreting our findings. In our study, we determined only six variants that may not cover the whole *FKBP5* gene. It is likely that genome-wide association studies would provide more comprehensive insight into the effects of variation in the *FKBP5* and its interaction with variants in other genes. Some of them did not follow the HWE, suggesting that representativeness of the sample might be limited. Secondly, the proportion of variance in the level of PLEs was also relatively low, suggesting that other factors not recorded by our study might be associated with PLEs. These factors might include familial liability for psychosis, depressive and anxiety symptoms and the level of perceived stress. Third, the data collected from the participants were based on self-reports, which might be characterized by a recall bias. However, reliability of trauma self-reports has been found to be stable over time in patients with psychosis [70]. Another limitation is that our sample had limited size, and independent replication of our findings was not performed. Moreover, this study was based on a non-clinical population, and thus generalization of findings cannot be made. Finally, a cross-sectional design does not allow for making conclusions on causal associations. Nevertheless, it is important to highlight that in contrary to multiple previous studies investigating the role of childhood trauma in the development of psychosis, this study focused also on cumulative lifetime traumatic experiences. To date, the body of studies on adulthood trauma in association with psychosis is very poor. It has previously been reported that TLEs in adulthood may have a different influence on the development of psychosis than childhood trauma. For instance, the study by Liu et al. observed that traumatic events occurring in the adulthood are associated with more severe positive symptoms in patients with schizophrenia, whereas childhood trauma is rather related to more severe depressive symptoms [71].

## 5. Conclusions

The present study indicates that variation in the *FKBP5* gene also moderates the effects of lifetime traumatic events on psychosis proneness. These findings provide grounds for developing more personalized approaches in predicting the outcomes of TLEs and selecting interventions that aim to restore psychological well-being in this population. However, before their application, larger longitudinal studies that combine results of genetic testing based on high throughput technologies with detailed assessment of complex psychological processes mediating the association between traumatic life events and psychosis are needed.

## Figures and Tables

**Table 1:** General characteristics of the sample.

	*n*	Mean ± SD or *n* (%)
Age, years	461	23.4 ± 3.0
Gender, M/F	460	133/327 (40.7/59.3)
Clinical diagnosis	461	38 (8.2)
EN	461	157 (34.1)
EA	461	191 (41.4)
PA	461	71 (15.4)
SA	461	38 (8.2)
PQ-16	461	4.1 ± 4.6
rs1360780	444	
CC		260 (58.56)
CT		159 (3.46)
TT		31 (6.98)
rs9296158	445	
AA		26 (5.84)
AG		159 (35.73)
GG		260 (58.43)
rs3800373	443	
GG		37 (8.35)
TG		144 (32.51)
TT		262 (59.14)
rs9470080	443	
CC		245 (55.30)
CT		151 (34.09)
TT		47 (10.61)
rs4713902	441	
CC		50 (11.34)
CT		154 (34.92)
TT		237 (53.74)
rs737054	449	
CC		224 (49.89)
CT		182 (40.53)
TT		43 (9.58)

Abbreviations: TEC, Traumatic Events Checklist; EN, emotional neglect; EA, emotional abuse; PA, physical abuse; SA, sexual abuse; PQ-16, the Prodromal Questionnaire 16.

**Table 2:** The PQ-16 score with respect to a history of TLEs.

	TLEs (+)	TLEs (-)	*p*
EA	5.10 ± 5.25	3.41 ± 3.81	<0.001
EN	5.25 ± 5.46	3.52 ± 3.91	<0.001
PA	5.24 ± 5.69	3.90 ± 4.30	0.022
SA	7.10 ± 6.76	3.83 ± 4.22	<0.001

Abbreviations: TLEs(+), positive history of traumatic life events; TLEs(-), negative history of traumatic life events; EN, emotional neglect; EA, emotional abuse; PA, physical abuse; SA, sexual abuse.

**Table 3:** Main and interactive effects of the FKBP5 variants on the PQ-16 score.

TLEs	IV or Covariate	rs1360780	rs9296158	rs3800373	rs9470080	rs4713902	rs737054
EN	Age	*F* = 48.00, *p* < 0.001	*F* = 47.28, *p* < 0.001	*F* = 43.37, *p* < 0.001	*F* = 42.87, *p* < 0.001	*F* = 41.92, *p* < 0.001	*F* = 45.36, *p* < 0.001
	Gender	*F* = 1.62, *p* = 0.204	*F* = 1.67, *p* = 0.197	*F* = 1.59, *p* = 0.208	*F* = 1.76, *p* = 0.186	*F* = 1.55, *p* = 0.214	*F* = 2.20, *p* = 0.139
	TLEs	*F* = 15.48, *p* < 0.001	*F* = 14.54, *p* < 0.001	*F* = 1.60, *p* = 0.207	*F* = 0.33, *p* = 0.564	*F* = 16.48, *p* < 0.001	*F* = 17.54, *p* < 0.001
	*FKBP5*	*F* = 1.15, *p* = 0.285	*F* = 1.96, *p* = 0.162	*F* = 3.24, *p* = 0.073	*F* = 1.07, *p* = 0.301	*F* = 0.24, *p* = 0.627	*F* = 0.19, *p* = 0.660
	*FKBP5* × TLEs	*F* = 1.04, *p* = 0.309	*F* = 1.21, *p* = 0.273	*F* = 1.43, *p* = 0.232	*F* = 5.35, *p* = 0.21	*F* = 2.45, *p* = 0.118	*F* = 7.84, *p* = 0.005
	R^2^	0.142	0.141	0.150	0.144	0.134	0.141
EA	Age	*F* = 48.84, *p* < 0.001	*F* = 47.94, *p* < 0.001	*F* = 43.58, *p* < 0.001	*F* = 42.56, *p* < 0.001	*F* = 44.25, *p* < 0.001	*F* = 45.82, *p* < 0.001
	Gender	*F* = 2.48, *p* = 0.116	*F* = 2.53, *p* = 0.112	*F* = 2.20, *p* = 0.139	*F* = 2.14, *p* = 0.145	*F* = 2.22, *p* = 0.137	*F* = 2.67, *p* = 0.103
	TLEs	*F* = 20.13, *p* < 0.001	*F* = 19.35, *p* < 0.001	*F* = 2.55, *p* = 0.111	*F* = 2.50, *p* = 0.115	*F* = 19.79, *p* < 0.001	*F* = 19.57, *p* < 0.001
	*FKBP5*	*F* = 0.10, *p* = 0.747	*F* = 0.48, *p* = 0.491	*F* = 4.64, *p* = 0.032	*F* = 2.45, *p* = 0.119	*F* = 1.89, *p* = 0.170	*F* = 0.67, *p* = 0.414
	*FKBP5* × TLEs	*F* = 0.12, *p* = 0.731	*F* = 0.06, *p* = 0.815	*F* = 1.12, *p* = 0.290	*F* = 2.18, *p* = 0.141	*F* = 0.11, *p* = 0.739	*F* = 0.832, *p* = 0.362
	R^2^	0.147	0.145	0.155	0.147	0.138	0.143
PA	Age	*F* = 49.06, *p* < 0.001	*F* = 48.56, *p* < 0.001	*F* = 42.46, *p* < 0.001	*F* = 41.35, *p* < 0.001	*F* = 41.52, *p* < 0.001	*F* = 45.54, *p* < 0.001
	Gender	*F* = 4.43, *p* = 0.038	*F* = 4.07, *p* = 0.044	*F* = 3.59, *p* = 0.059	*F* = 3.33, *p* = 0.069	*F* = 3.15, *p* = 0.077	*F* = 3.79, *p* = 0.052
	TLEs	*F* = 8.87, *p* = 0.003	*F* = 7.44, *p* = 0.007	*F* = 1.83, *p* = 0.177	*F* = 2.17, *p* = 0.142	*F* = 5.78, *p* = 0.017	*F* = 8.305, *p* = 0.004
	*FKBP5*	*F* = 4.12, *p* = 0.43	*F* = 5.90, *p* = 0.016	*F* = 1.95, *p* = 0.164	*F* = 1.23, *p* = 0.269	*F* = 0.90, *p* = 0.343	*F* = 0.12, *p* = 0.729
	*FKBP5* × TLEs	*F* = 5.53, *p* = 0.019	*F* = 6.80, *p* = 0.009	*F* = 0.10, *p* = 0.752	*F* = 0.01, *p* = 0.925	*F* = 0.00, *p* = 0.927	*F* = 1.88, *p* = 0.171
	R^2^	0.141	0.141	0.131	0.121	0.111	0.125
SA	Age	*F* = 45.09, *p* < 0.001	*F* = 44.19, *p* < 0.001	*F* = 39.66, *p* < 0.001	*F* = 39.19, *p* < 0.001	*F* = 40.52, *p* < 0.001	*F* = 42.56, *p* < 0.001
	Gender	*F* = 1.33, *p* = 0.250	*F* = 1.42, *p* = 0.235	*F* = 1.31, *p* = 0.252	*F* = 1.35, *p* = 0.245	*F* = 1.38, *p* = 0.242	*F* = 1.57, *p* = 0.211
	TLEs	*F* = 16.78, *p* < 0.001	*F* = 15.05, *p* < 0.001	*F* = 5.95, *p* = 0.015	*F* = 6.70, *p* = 0.010	*F* = 11.50, *p* = 0.001	*F* = 17.68, *p* < 0.001
	*FKBP5*	*F* = 1.19, *p* = 0.275	*F* = 0.96, *p* = 0.329	*F* = 1.05, *p* = 0.305	*F* = 1,79, *p* = 0.182	*F* = 1.47, *p* = 0.227	*F* = 0.18, *p* = 0.671
	*FKBP5* × TLEs	*F* = 0.93, *p* = 0.337	*F* = 0.37, *p* = 0.543	*F* = 0.39, *p* = 0.531	*F* = 0.19, *p* = 0.732	*F* = 0.38, *p* = 0.538	*F* = 0.93, *p* = 0.336
	R^2^	0.146	0.141	0.145	0.129	0.125	0.139

Abbreviations: IV, independent variable; TLEs, traumatic life events; EN—emotional neglect; EA—emotional abuse; PA—physical abuse; SA—sexual abuse, PQ-16—Prodromal Questionnaire 16.

## Data Availability

The data presented in this study are available on request from the corresponding author.
